# The effect of remote health intervention based on internet or mobile communication network on hypertension patients: Protocol for a systematic review and meta-analysis of randomized controlled trials

**DOI:** 10.1097/MD.0000000000014707

**Published:** 2019-03-01

**Authors:** Yong Wu, Pei Zhao, Wei Li, Ming-Qiang Cao, Lin Du, Jian-Chang Chen

**Affiliations:** a Department of Cardiology, the Second Affiliated Hospital of Soochow University; b Department of Cardiology, the Affiliated Hospital of Yangzhou University, Hanjiang District, Yangzhou; c Department of Cardiology, the Frist Affiliated Hospital of Soochow University, Gusu District, Suzhou, Jiangsu, China.

**Keywords:** internet, meta-analysis, mobile communication network, randomized controlled trials, remote health intervention, systematic review

## Abstract

**Background:**

To systematically review the impact of remote health interventions based on an internet or mobile communication network on patients with hypertension and to provide a theoretical basis for hypertension patients with the implementation of remote health interventions.

**Methods:**

Data were retrieved from a total of 4 Chinese databases and 3 foreign databases. The Chinese databases included: China National Knowledge Infrastructure (CNKI), WanFang Data, Chinese Biomedical Database (SinoMed), and Chongqing Chinese Science and Technology Journey database (VIP). The foreign language databases included PubMed, The Cochrane Library, and EMbase, and the date range for the search was from the date the database became active to December 1^st^, 2018. After screening and extracting the materials and evaluating the risk of bias in each study (conducted by 2 researchers), the quality of the selected literature was evaluated by Review Manager (RevMan) [Computer program]. Version 5.3. Copenhagen: The Nordic Cochrane Centre, The Cochrane Collaboration, 2014, and the statistical analysis was applied by Stata 12.0 software.

**Result:**

This study will provide high-quality evidence-based medicine research evidence for remote health interventions on hypertensive patients based on the Internet and mobile communication network using systematic evaluation and meta-analysis methods.

**Conclusion:**

This systematic review will provide a scientific conclusion as to whether the remote health intervention model based on an internet or mobile communication network can better control blood pressure and improve patient compliance than the traditional nursing intervention model for hypertensive patients.

**Ethics and dissemination:**

This protocol for a systematic review and meta-analysis of randomized controlled trials does not require ethical approval and the results of this paper will be published in an open form in internationally influential academic journals.

**Protocol and registration:**

A protocol had been registered in PROSPERO CRD42019122404.

## 1 Introduction

Cardiovascular disease is the leading cause of death due to noncommunicable disease. Hypertension, one of the major risk factors of cardiovascular disease, is a common chronic disease that has become a global public health problem. At present, it is estimated that >1.5 billion people worldwide are suffering from hypertension.^[1]^ With the rapid development of the social economy, the acceleration of population aging, and the change of traditional dietary habits and lifestyle, the incidence of prehypertension in the world has significantly increased. For example, approximately 31% of the population in the United States are in the prehypertension range.^[2]^ A national survey in 2014 showed that the control rate of hypertension was 9.3%. More than 70% of the patients receiving antihypertensive treatment did not meet the blood pressure standard.^[3]^ That study showed that hypertension is a disease that can be prevented and controlled. Reducing the blood pressure level of patients can reduce the incidence of stroke and cardiovascular events, significantly improve the patient's quality of life, and effectively reduce the burden of disease. At present, health management is a relatively mature and effective way to address chronic diseases, such as hypertension. Health management is the process of health consultation and guidance provided by health managers after comprehensive analysis of the health risk factors for assessment of the individual or group. Through long-term health management, the hypertension rate in the United States increased from 10% to 28% between 1976 and 2006. By promoting health management, Finland changed people's lifestyle to reduce cholesterol, blood pressure levels, and ultimately, the mortality rate of cardiovascular diseases in the country.^[4]^ At the same time, the health expenditure can be effectively controlled by investing in health management.

In recent years, with the continuous development and popularization of the internet and mobile communication network, information technology has been increasingly applied to blood pressure management. Since some hypertensive patients need long-term medication for treatment, blood pressure should be monitored while taking the medication, and attention should be paid to the adjustment of bad lifestyle factors, so health education has become an important part of continuous treatment for patients after discharge. In addition, previous studies have shown that patients will gradually forget the advice given by the medical staff, and their compliance with drugs will decrease with the passage of time after discharge.^[5]^ Continuous health intervention for discharged patients based on an internet and mobile communication network can not only save medical resources but also determine the existing problems of patients with no delay, as well as the patients’ compliance with drugs. Therefore, medical staff can carry out remote health interventions for hypertension patients using several of these methods, including the internet and mobile communication network platform, telephone follow-up, short message reminder on a mobile phone, and network education. Health education carried out through internet and mobile communication networks solves the problems of long space distance in big cities and inconvenient transportation between urban and rural areas and communities, which lead to difficulty in patients seeking medical treatment, and these educational methods are more flexible and diverse.^[6]^ Therefore, this study used a meta-analysis method to conduct a comprehensive analysis of the relevant research at home and abroad, aiming to evaluate the effect of remote health interventions on hypertensive patients based on the internet and mobile communication network.

## 2 Methods

### 2.1 Protocol and registration

A protocol had been registered in PROSPERO CRD42019122404.

### 2.2 Criteria for included studies

#### 2.2.1 Types of studies

The studies were randomized controlled trial using internet or mobile communication network tools for remote health interventions for hypertension patients. No language restrictions were imposed, whether blind or not. This study is based on the systematic review and meta-analysis protocols (PROSMA-P) guidance.^[7]^

#### 2.2.2 Types of participants

Types of participant: inclusion criteria^[8]^: publicly published Chinese literature with complete data provided; all subjects met the diagnostic criteria for hypertension, that is, systolic blood pressure ≥140 and/or diastolic blood pressure ≥90 mmHg (1 mmHg = 0.133 kPa); study was a randomized controlled trial, whether or not a blind method was mentioned; clear outcome indicators were included; and the patient's age, sex, source of case, course of disease, and type of hypertension were not limited.

Exclusion criteria: non-randomized controlled trial (RCT) literature; inconsistent baseline data; no specific intervention time; treatment measures did not meet the preselection criteria; nonclinical research literature, such as animal experiments, reviews, individual case reports, etc.; and repeated published literature.

#### 2.2.3 Types of interventions

Interventions: experimental group (remote health intervention, RHI): health education telephone follow-up and short message reminder (provided by computer software) was conducted for hypertension patients using internet or mobile communication network tools; control group (traditional nursing interventions, TNI): routine health education, social natural education, or patients’ active learning of health content through network platform was adopted.

#### 2.2.4 Outcome measures

Primary outcomes: systolic blood pressure (mm Hg) and diastolic blood pressure (mm Hg); knowledge of prevention and treatment of hypertension and its hazards; secondary outcomes: patients’ compliance: medication compliance; regular blood pressure measurement; reasonable diet; quitting smoking and alcohol restriction; weight control; sticking to appropriate exercise; sticking to regular review; regular life patterns.

### 2.3 Search strategy and study selection

#### 2.3.1 Database selection

Network databases, including foreign databases PubMed, The Cochrane Library, and EMbase, and domestic databases, such as CNKI, WangFang Date, SinoMed, and VIP, were searched.

#### 2.3.2 Search strategy

The retrieval scheme was to mainly adopt the combination of subject words and free words, among which, the Chinese search terms were “Gao-xue-ya,” “Jian-kang-jiao-yu,” “Jian-kang-zhi-dao,” “Jian-kang-gan-yu,” “Yuan-cheng-jiao-yu,” “Dian-zi-zi-xun,” “Dian-hua-sui-fang,” “Yi-dong-wang-luo,” “Dian-hua,” “Shou-ji,” “Hu-lian-wang,” “Shou-ji-duan-xin,” “Wei-xin,” “ICQ,” and “QQ,” etc.; the English search terms were “Hypertension,” “Blood pressure,” “Telemedicine,” “Web-based intervention,” “Mobile network,” “Telephone,” “Mobile phone,” “internet,” “Short message,” “WeChat,” “Health Education,” and “Distance Counseling,” etc. The search language was not restricted, and the search scope was set from the establishment of the database to December 1^st^, 2018. The papers and periodicals with no electronic editions after 2010 were manually retrieved. The search strategy is provided in Table 1 .

**Table 1:**
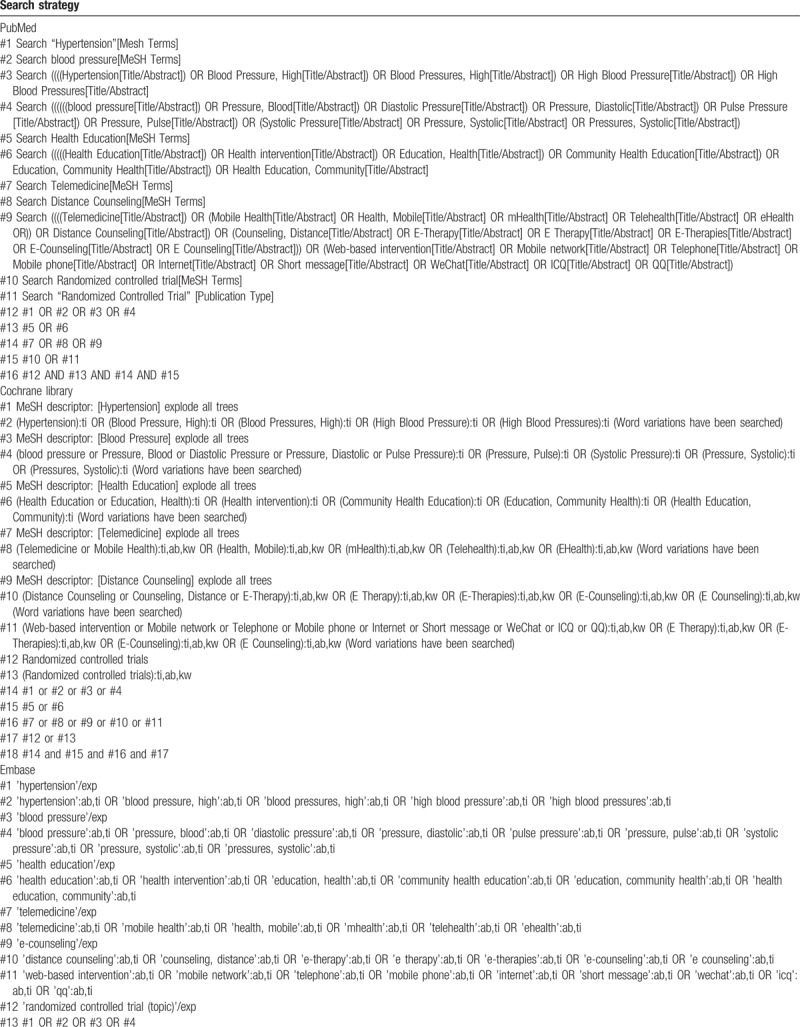
Search strategy for the 7 electronic databases.

**Table 1 (Continued):**
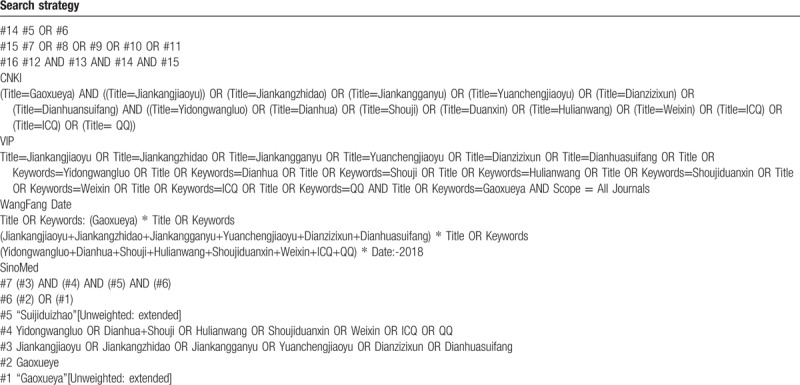
Search strategy for the 7 electronic databases.

### 2.4 Data collection and analysis

#### 2.4.1 Data extraction

**Figure 1:**
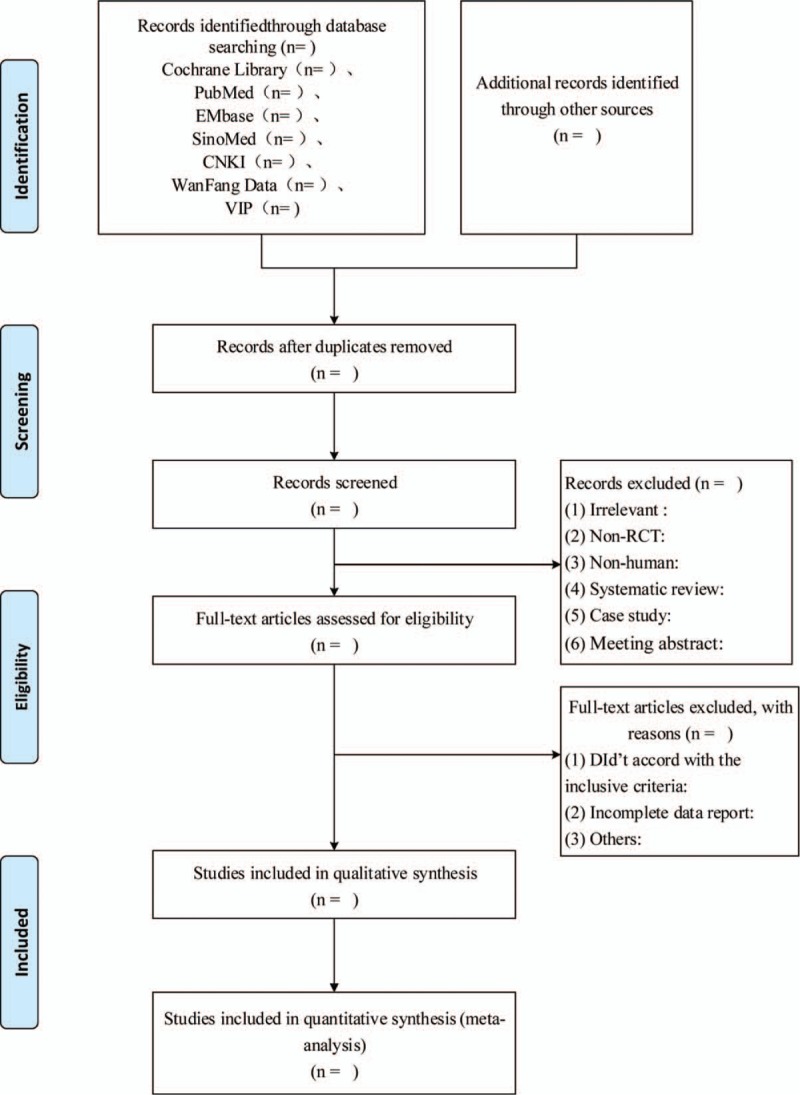
Screening flow chart for selected literature.

According to the preferred reporting items for systematic reviews and meta-analyses flow chart, 2 researchers (ZP and CM) independently screened the literature according to the inclusion/exclusion criteria and checked each other. The dissenting literature was discussed and solved by a third researcher. A predesigned Excel data extraction table was used to extract information, including: basic information included in the study; basic information about the study subjects; relevant information about the experiment and the control group (i.e., sample size, course of treatment, baseline data, and treatment methods of the 2 groups); outcome indicators; quality evaluation of the literature, etc. The screening flow chart of the literature, please see Fig. 1.

#### 2.4.2 Assessment of risk of bias

The Quality Assessment was based on the Risk of Bias Tool of the “Cochrane Handbook for Systematic Assessment of Interventions” and the relevant regulations of the Assessment Guidelines and was performed by selected researchers to assess the risk of bias in the included literature.^[9]^ There were 7 main programs for the tools of risk assessment: random sequences generation; the concealing allocation schemes; the adoption of blind methods by both implementers and participants; the adoption of blind methods by outcome evaluators; incomplete outcome data; selective publication; and the remaining bias. According to the evaluation criteria, 7 projects were evaluated as “high bias risk (H),” “low bias risk (L),” and “unknown bias risk (U).”

#### 2.4.3 Data synthesis

Meta-analysis software Stata 12.0 (StataCorp LLC, Texas) was used to conduct the statistical analysis. Selection of effect variables: if the index for testing the results of the included literature was a two-class variable, then the statistic of efficacy analysis was represented by relative risk, which was expressed by a 95% confidence interval; 95% confidence interval with weighted mean difference was used to represent continuity change. Heterogeneity testing: a step to test statistical results for homogeneity, that is, to test the degree of variation of various original research results and to explicitly clarify the homogeneity of the test. Meta-analysis: according to the results of heterogeneity test, when *P* ≥ .05, *I*^2^ < 50, which indicated that the results were consistent and could be analyzed by a fixed-effect model. If *P* < .05, *I*^2^ ≥ 50, which indicated the heterogeneity of the results could not be neglected. If the clinical significance of the included study still existed, random effect model could be used.

#### 2.4.4 Sensitivity analysis

Meta-analysis synthesizes multiple results, excludes possible anomalies, re-evaluates them and compares the results with those of pre-excluded meta-analysis in order to study the possible impact of excluded studies on the merger effect index and the stability of the meta-analysis results. If there was little difference between the two results, the low sensitivity of the results was stable and there was high reliability.^[10]^

#### 2.4.5 Subgroup analysis and solutions to heterogeneity

If the heterogeneity of a certain outcome is very large, the subgroup analysis should be carried out according to the actual circumstances of the study and the possible influencing factors.^[11]^ This study analyzed the impact of remote health interventions on hypertension patients based on an internet or mobile communication network. Therefore, the possible factors, such as duration of intervention, frequency of intervention, and source of patients, may have been the reasons for the increase in outcome indicator heterogeneity. The factors that may cause heterogeneity were grouped according to the results of the heterogeneity test to reduce the source of heterogeneity. If the heterogeneity between the studies was too large or it was impossible to find data sources, only a descriptive analysis was used.

### 2.5 Measures for publication bias

Publication bias occurs because positive data in papers containing statistical significance in similar studies are more likely to be accepted and published by journals, which is difficult to control. In this study, Stata 12.0 software was used to identify potential publication bias. When the number of studies selected in the outcome indicators was ≥8, Egger test was used to detect the publication bias of the relevant indicators, and funnel maps were drawn at the same time. If there was publication bias, a nonparametric clipping method was used to estimate the impact of publication bias on the results of the study.

### 2.6 Ethics and dissemination

The data used in this study are derived from relevant data in published academic papers and this protocol for a systematic review and meta-analysis of randomized controlled trials does not require ethical approval and the results of this paper will be published in an open form in internationally influential academic journals.

## 3 Discussion

Hypertension is the most common chronic, noncommunicable disease, and it is also the disease with the largest burden in the world, requiring systematic management and lifelong treatment.^[12,13]^ Individual treatment plans should be formulated according to the actual circumstances of individual patients. It is not enough to rely on the diagnosis and treatment during hospitalization alone. More detailed management work, such as registration and filing, regular follow-up, and health education, should be implemented and completed with the help of nearby medical units, namely, community service centers (stations). Approximately 90% of hypertensive patients see doctors in rural or urban primary medical institutions, and community outpatient clinics will be the main battlefield of hypertension prevention and treatment.^[14]^ Therefore, it is necessary to attach importance to and strengthen the health management ability of community patients via health promotion and health management and by taking prevention-based and prevention-combined approaches to community health management for hypertension. There are, however, 2 difficulties at present^[15]^:the existing management model belongs to the one-way management model of doctors to patients, which lacks the active participation of patients and the active interaction between patients and doctors without carrying out the individual health management interventions for different situations, while the community contracted service is limited by community medical resources with limited coverage; and once diagnosed, the vast majority of hypertensive patients need lifelong medication. Drug selection is very important, but more importantly, the compliance of patients is directly related to the efficacy and prognosis. According to the results of an epidemiological survey of hypertension in China, the control rate of hypertension in China only accounts for 8.3%.^[16]^ One of the key reasons is poor patients’ compliance. Therefore, remote health intervention based on internet or mobile communication networks may be a key factor in addressing these issues in the health management service model for hypertensive patients.

Remote health interventions based on internet or mobile communication networks can improve patients’ compliance behavior. Compliance behavior is patients’ compliance, which refers to the degree of compliance between patients’ behaviors (e.g., medication, diet, behavior mode) and clinical doctor's advice after seeking medical treatment. A patient's compliance with treatment is the basis of effective treatment. Low compliance is a common phenomenon among discharged patients or outpatients. Discharged patients and outpatient patients can only remember 50% to 60% of the discharge guidance of nurses. With the passage of time, the instructions of doctors and nurses will gradually fade away, and patients’ compliance will decrease^[17,18]^; however, regular remote health interventions can remind and help patients to take medicine and maintain a healthy lifestyle, especially elderly patients and hypertensive patients in remote rural areas. The remote health intervention model based on internet or mobile communication networks is a form of health education worthy of promotion. It is an open and extended health education with the change of medical model. The remote health intervention model is an effective means to extend hospital health education to patients’ homes. Intervention measures provided by internet or mobile communication network platforms can guide and monitor patients’ condition changes, psychological status, and health status, and it can establish communication channels of medical care-related information in a timely and effective manner.

The purpose of this study was to compare the advantages and disadvantages of remote health interventions and traditional nursing interventions in controlling blood pressure and compliance in patients with hypertension. The results of the study will help patients with hypertension who have been discharged from hospital or community to effectively control blood pressure, and also provide scientific evidence and theoretical guidance for the prevention and control of hypertension.

## Author contributions

Jian-Chang Chen and Yong Wu designed the study. Yong Wu, Pei Zhao, and Ming-Qiang Cao collected the literatures. Wei Li, Lin Du, and Yong Wu carried out the extraction and sorting of the basic data of the literatures. Jian-Chang Chen and Yong Wu set the statistical methods used in this study. Pei Zhao and Ming-Qiang Cao used software to perform statistical calculations. Yong Wu, Pei Zhao, Ming-Qiang Cao, Wei Li, and Lin Du participated in the drafting of the first draft, and Jian-Chang Chen reviewed the paper and registered a protocol on PROSPERO.

**Data curation:** Jian-Chang Chen, Yong Wu.

**Formal analysis:** Yong Wu, Pei Zhao, Ming-Qiang Cao, Wei Li, Lin Du.

**Investigation:** Yong Wu, Pei Zhao, Ming-Qiang Cao.

**Methodology:** Jian-Chang Chen, Yong Wu.

**Project administration**: Jian-Chang Chen.

**Resources:** Jian-Chang Chen.

**Software:** Pei Zhao, Ming-Qiang Cao.

**Supervision:** Jian-Chang Chen.

**Writing – original draft:** Yong Wu, Pei Zhao, Ming-Qiang Cao, Wei Li, Lin Du.

**Writing – review & editing**: Jian-Chang Chen.

Jian-Chang Chen orcid: 0000-0003-1090-5404.

## References

[1] Huo YR Analysis of epidemiological characteristics and risk factors of hypertension in community elderly people. Nurs Pract Res 2018;15:26–8.

[2] Mendis S O’Brien E Seedat YK Hypertension and diabetes: entry points for prevention and control of the global cardiovascular epidemic. Int J Hypertens 2013;2013:878460. 23653856 10.1155/2013/878460 PMC3638673

[3] Lee CG The emerging epidemic of hypertension in Asian children and adolescents. Curr Hypertens Rep 2014;16:495. 25304106 10.1007/s11906-014-0495-z

[4] da Silva FM Budó Mde L Girardon-Perlini NM Contributions of health education groups to the knowledge of people with hypertension. Rev Bras Enferm 2014;67:347–53. 25054694 10.5935/0034-7167.20140045

[5] Wang YL Zhang L Application of telephone follow-up health education guidance in patients with hypertension discharged from hospital. China Pract Med 2014;9:262–3.

[6] Bosworth HB Olsen MK Gentry P Nurse administered telephone intervention for blood pressure control: a patient-tailored multifactorial intervention. Patient Educ Couns 2005;57:5–14. 15797147 10.1016/j.pec.2004.03.011

[7] Shamseer L Moher D Clarke M PRISMA-P Group. Preferred reporting items for systematic review and meta-analysis protocols (PRISMA-P) 2015: elaboration and explanation. BMJ 2015;350:7647. 10.1136/bmj.g7647 25555855

[8] Wen J Hua Q Chen WP The impact of diagnosis and treatment for newly hypertensive patients according to ACC/AHA guideline for hypertension. J Clin Cardiol 2018;34:1136–8.

[9] Jordan VM Lensen SF Farquhar CM There were large discrepancies in risk of bias tool judgments when a randomized controlled trial appeared in more than one systematic review. J Clin Epidemiol 2017;81:72–6. 27622779 10.1016/j.jclinepi.2016.08.012

[10] Guan X Li HC Yao JQ Comparison and selection of application methods of meta-analysis results in economic evaluations. Chin J Evid Based Med 2018;18:1224–31.

[11] Zhang SH Subgroup analysis and sensitive analysis should be set up reasonably in meta-analysis. Chin J Contemp Neurol Neurosurg 2016;16:1–2.

[12] Lai M Tong H Wan Y The variance of hypertension prevalence detected by epidemiological survey against clinical practice: data from a rural population in South China. J Am Soc Hypertens 2018;12:103–6. 10.1016/j.jash.2018.11.006 30509779

[13] Yang XH Zhang WX Shuai W The clinical diagnosis rate of hypertension in the subjects with newly-diagnosed hypertension identified by epidemiological study. Chin J Hypertens 2018;26:1026–9.

[14] Wu CF Zhu L Hu HP Application effect of community health education in patients with essential hypertension. Med Innovat China 2018;15:73–6.

[15] Zhu YY Ren JP Li ST Research of PRECEDE model impact on community hypertension patients’health behaviors. Med Philos 2017;38:42–5.

[16] Zhang YF Ye H Luo FF Research progress of community hypertension management model. Shenzhen J Integr Trad Chin Western Med 2018;28:190–3.

[17] Yin BD Analysis of influencing factors and intervention strategies on compliance of hypertension drug treatment in community. Chin J Evid Based Cardiovasc Med 2017;9:991–4.

[18] Green BB Cook AJ Ralston JD Effectiveness of home blood pressure monitoring, web communication, and pharmacist care on hypertension control: a randomized controlled trial. JAMA 2008;299:2857–67. 18577730 10.1001/jama.299.24.2857 PMC2715866

